# Crystal structure of (*E*)-2-[1-(benzo[*d*][1,3]dioxol-5-yl)ethyl­idene]-*N*-methyl­hydrazine-1-carbo­thio­amide

**DOI:** 10.1107/S2056989014026395

**Published:** 2015-01-01

**Authors:** Adriano Bof de Oliveira, Christian Näther, Inke Jess, Renan Lira de Farias, Iasmin Alves Ribeiro

**Affiliations:** a Departamento de Química, Universidade Federal de Sergipe, Av. Marechal Rondon s/n, 49100-000 São Cristóvão-SE, Brazil; b Institut für Anorganische Chemie, Christian-Albrechts-Universität zu Kiel, Max-Eyth Strasse 2, D-24118 Kiel, Germany

**Keywords:** crystal structure, thio­semiarbazone, 3′,4′-(methyl­enedi­oxy)aceto­phenone, 4-methyl­thio­semicarbazone, hydrogen bonding, two-dimensional network

## Abstract

In the title compound, C_11_H_13_N_3_O_2_S, there is a short intra­molecular N—H⋯N contact. The benzo[*d*][1,3]dioxole ring system is approximately planar (r.m.s. deviation = 0.025 Å) and makes a dihedral angle of 56.83 (6)° with the mean plane of the methyl­thio­semicarbazone fragment [–N—N—C(=S)—N—C; maximum deviation = 0.1111 (14) Å for the imino N atom]. In the crystal, mol­ecules are linked *via* pairs of N—H⋯S hydrogen bonds, forming inversion dimers. The dimers are connected by N—H⋯S hydrogen bonds into layers parallel to (100). The H atoms of both methyl groups are disordered over two sets of sites and were refined with occupancy ratios of 0.5:0.5 and 0.75:0.25.

## Related literature   

For one of the first reports of the synthesis of thio­semicarbazone derivatives, see: Freund & Schander (1902 ▸). For one of the first reports of 3,4-methyl­ene­dioxy­aceto­phenone and its extraction from the South American rosewood tree, see: Mors *et al.* (1957 ▸). For the crystal structure of a derivative of the title compound, 1-(2*H*-1,3-benzodioxol-5-yl)ethanone thio­semicarbazone, see: Oliveira *et al.* (2013 ▸).
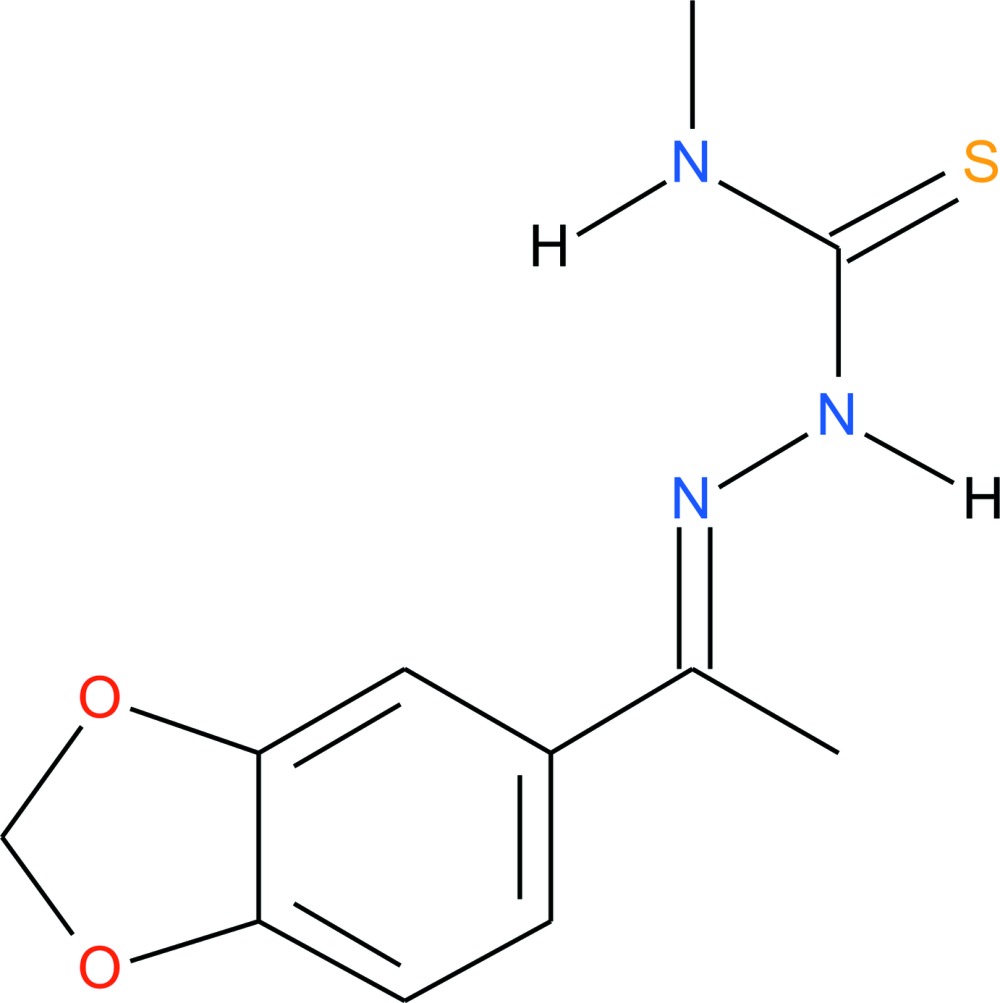


## Experimental   

### Crystal data  

C_11_H_13_N_3_O_2_S
*M* *_r_* = 251.30
Monoclinic, 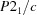

*a* = 8.7927 (4) Å
*b* = 12.5979 (6) Å
*c* = 10.9254 (4) Å
β = 106.098 (3)°
*V* = 1162.75 (9) Å^3^
*Z* = 4
Mo *K*α radiation
μ = 0.27 mm^−1^
*T* = 200 K
0.2 × 0.1 × 0.1 mm

### Data collection  

Stoe IPDS-1 diffractometer
12631 measured reflections
2530 independent reflections
2166 reflections with *I* > 2σ(*I*)
*R*_int_ = 0.034

### Refinement  

*R*[*F* ^2^ > 2σ(*F* ^2^)] = 0.038
*wR*(*F* ^2^) = 0.096
*S* = 1.06
2530 reflections
157 parameters
H-atom parameters constrained
Δρ_max_ = 0.22 e Å^−3^
Δρ_min_ = −0.21 e Å^−3^

### 

Data collection: *X-AREA* (Stoe & Cie, 2008 ▸); cell refinement: *X-AREA*; data reduction: *X-RED32* (Stoe & Cie, 2008 ▸); program(s) used to solve structure: *SHELXS97* (Sheldrick, 2008 ▸); program(s) used to refine structure: *SHELXL97* (Sheldrick, 2008 ▸); molecular graphics: *DIAMOND* (Brandenburg, 2006 ▸); software used to prepare material for publication: *SHELXL97*, *PLATON* (Spek, 2009 ▸) and *publCIF* (Westrip, 2010 ▸).

## Supplementary Material

Crystal structure: contains datablock(s) I, publication_text. DOI: 10.1107/S2056989014026395/su5032sup1.cif

Structure factors: contains datablock(s) I. DOI: 10.1107/S2056989014026395/su5032Isup2.hkl

Supporting information file. DOI: 10.1107/S2056989014026395/su5032Isup3.cml

**Fig. 1.**
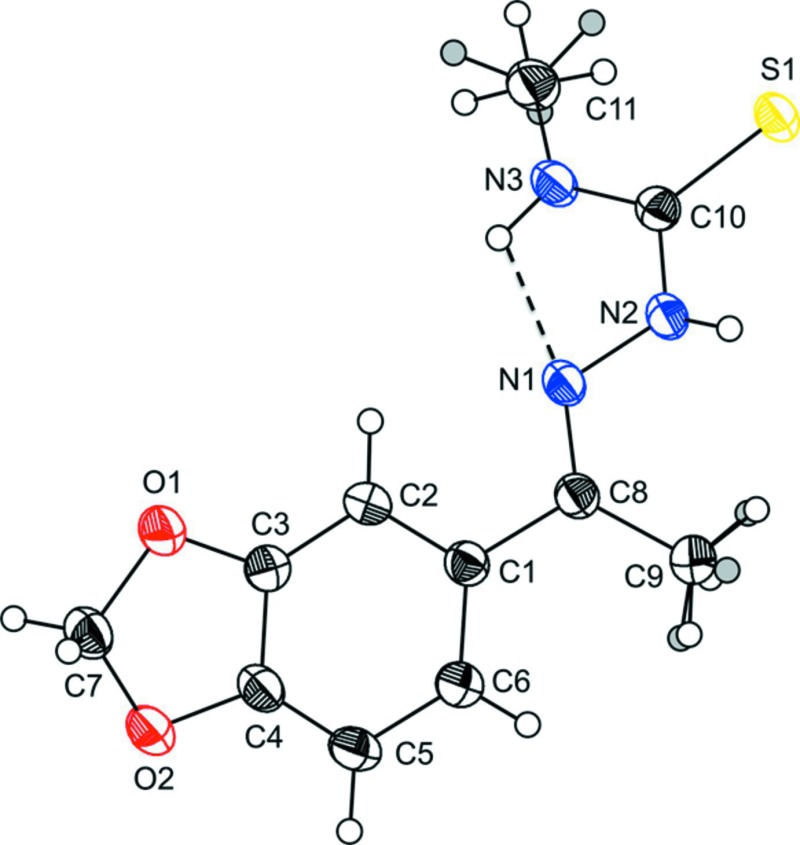
The molecular structure of the title compound with atom labelling. Displacement ellipsoids are drawn at the 40% probability level. Disordered H atoms are shown with white and light gray interior colours and the short intramolecular N-H···N contact is shown as a dashed line (see Table 1 for details).

. DOI: 10.1107/S2056989014026395/su5032fig1.tif
The mol­ecular structure of the title compound with atom labelling. Displacement ellipsoids are drawn at the 40% probability level. Disordered H atoms are shown with white and light gray inter­ior colours and the short intra­molecular N-H⋯N contact is shown as a dashed line (see Table 1 for details).

**Fig. 2.**
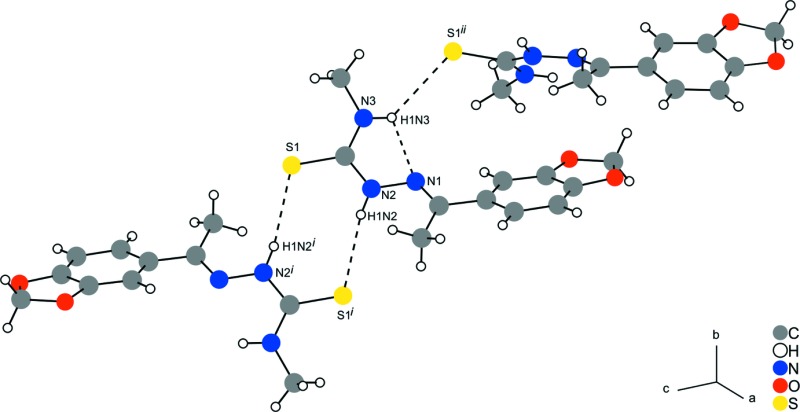
A view of the intramolecular and intermolecular hydrogen bonds (dashed lines) in the crystal structure of the title compound (see Table 1 for details of the hydrogen bonding and the symmetry codes; disordered H atoms are not shown for clarity).

**Fig. 3.**
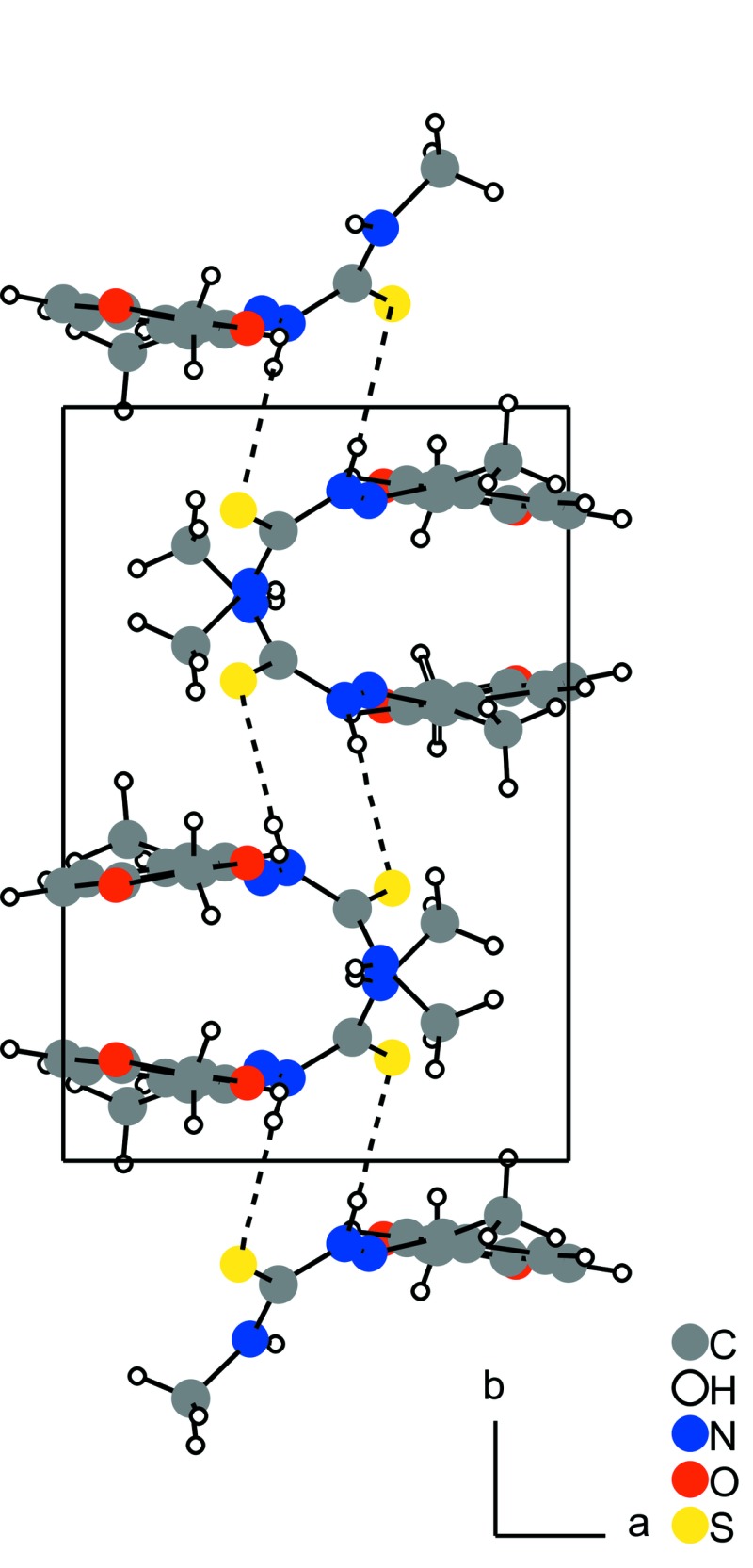
A partial view along the c axis of the crystal packing of the title compound. The N2—H1N2···S1 hydrogen bonds are shown as dashed lines (see Table 1 for details; disordered H atoms are not shown for clarity).

. DOI: 10.1107/S2056989014026395/su5032fig2.tif
A view of the intra­molecular and inter­molecular hydrogen bonds (dashed lines) in the crystal structure of the title compound (see Table 1 for details of the hydrogen bonding and the symmetry codes; disordered H atoms are not shown for clarity).

c H1N2 . DOI: 10.1107/S2056989014026395/su5032fig3.tif
A partial view along the *c* axis of the crystal packing of the title compound. The N2—*H1*N*2*⋯S1 hydrogen bonds are shown as dashed lines (see Table 1 for details; disordered H atoms are not shown for clarity).

CCDC reference: 1036961

Additional supporting information:  crystallographic information; 3D view; checkCIF report

## Figures and Tables

**Table 1:** Hydrogen-bond geometry (, )

*D*H*A*	*D*H	H*A*	*D* *A*	*D*H*A*
N3H1*N*3N1	0.88	2.17	2.6080(19)	110
N2H1*N*2S1^i^	0.88	2.62	3.4871(14)	168
N3H1*N*3S1^ii^	0.88	2.86	3.4973(14)	131

Symmetry codes: (i) 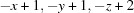; (ii) 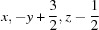.

## supplementary crystallographic information

### S1. Structural commentary

In our research we are inter­ested in the synthesis of thio­semicarbazone derivatives of natural products. Herein, we report the synthesis and crystal structure of 1-(2*H*-1,3-benzodioxol-5-yl)ethanone 4-methyl­thio­semicarbazone, a product of the reaction between 3',4'-(methyl­ene­dioxy)­aceto­phenone and 4-methyl­thio­semicarbazide. The ketone is a natural product obtained from the South American rosewood trees that belong to the *Lauraceae* family (Mors *et al.*, 1957).

In the title molecule, Fig. 1, the torsion angle for the N1–N2–C10–N3 entity is 10.2 (2)°. The maximum deviation from the mean plane of the non-H atoms for the C1—C9/O1—O2 fragment and for the C10—C11/N1—N3/S1 fragment amount to 0.2844 (14) Å and 0.1111 (12) Å, respectively, and the angle between their mean planes is 55.39 (4) °. The molecule has two disordered methyl groups. The H atoms of the terminal methyl substituent, C11, are disordered over two sets of sites with an occupancy ratio of 0.75:0.25, those of the other methyl substituent, C9, attached to the Schiff base are disordered over two sets of sites with an occupancy ratio of 0.5:0.5 (Fig. 1).

In the crystal, the molecules are linked *via* pairs of N2—*H1N2*···S1 hydrogen bonds into inversion dimers. These dimers are connected by weak N3—*H1N3*···S1 hydrogen bonds into layers, that are parallel to the *bc* plane. Finally, an intra­molecular N3—*H1N3*···N1 hydrogen bond is also observed (Figs. 2 and 3, and Table 1).

### S2. Synthesis and crystallization

The synthesis of the title compound was adapted from a previously reported procedure (Freund & Schander, 1902). In a hydro­chloric acid catalyzed reaction, a mixture of 3',4'-(methyl­ene­dioxy)­aceto­phenone (10 mmol) and 4-Methyl-3-thio­semicarbazide (10 mmol) in ethanol (80 ml) was refluxed for 6 h. After cooling and filtering, the title compound was obtained. Colourless crystals were obtained in DMSO by the slow evaporation of the solvent.

### S3. Refinement

Crystal data, data collection and structure refinement details are summarized in Table 2. The NH H atoms were located in a difference Fourier map and were refined as riding atoms with N—H = 0.88 Å and with *U*_iso_(H) = 1.5*U*_eq_(N). The C-bound H atoms were positioned with idealized geometry and refined as riding atoms: C—H = 0.95 - 0.99 Å with *U*_iso_(H) = 1.5*U*_eq_(C) for methyl H atoms and = 1.2U_eq_(C) for other H atoms. The H atoms of methyl groups, C9 and C11, are disordered over two positions and were refined in two different orientations rotated by 60° with occupancy ratios of 0.5:0.5 and 0.75:0.25, respectively.

### Crystal data

**Table:**

C_11_H_13_N_3_O_2_S	*Z* = 4
*M**_r_* = 251.30	*F*(000) = 528
Monoclinic, *P*2_1_/*c*	*D*_x_ = 1.436 Mg m^−^^3^
Hall symbol: -P 2ybc	Mo *K*α radiation, λ = 0.71073 Å
*a* = 8.7927 (4) Å	θ = 2.4–27.0°
*b* = 12.5979 (6) Å	µ = 0.27 mm^−^^1^
*c* = 10.9254 (4) Å	*T* = 200 K
β = 106.098 (3)°	Prism, colourless
*V* = 1162.75 (9) Å^3^	0.2 × 0.1 × 0.1 mm

### Data collection

**Table:**

Stoe IPDS-1 diffractometer	2166 reflections with *I* > 2σ(*I*)
Radiation source: fine-focus sealed tube, Stoe IPDS-1	*R*_int_ = 0.034
Graphite monochromator	θ_max_ = 27.0°, θ_min_ = 2.4°
φ scans	*h* = −11→11
12631 measured reflections	*k* = −16→16
2530 independent reflections	*l* = −13→13

### Refinement

**Table:**

Refinement on *F*^2^	Secondary atom site location: difference Fourier map
Least-squares matrix: full	Hydrogen site location: inferred from neighbouring sites
*R*[*F*^2^ > 2σ(*F*^2^)] = 0.038	H-atom parameters constrained
*wR*(*F*^2^) = 0.096	*w* = 1/[σ^2^(*F*_o_^2^) + (0.0452*P*)^2^ + 0.3583*P*] where *P* = (*F*_o_^2^ + 2*F*_c_^2^)/3
*S* = 1.06	(Δ/σ)_max_ < 0.001
2530 reflections	Δρ_max_ = 0.22 e Å^−^^3^
157 parameters	Δρ_min_ = −0.21 e Å^−^^3^
0 restraints	Extinction correction: *SHELXL97* (Sheldrick, 2008), Fc^*^=kFc[1+0.001xFc^2^λ^3^/sin(2θ)]^-1/4^
Primary atom site location: structure-invariant direct methods	Extinction coefficient: 0.012 (3)

### Special details

**Table:**

Geometry. All e.s.d.'s (except the e.s.d. in the dihedral angle between two l.s. planes) are estimated using the full covariance matrix. The cell e.s.d.'s are taken into account individually in the estimation of e.s.d.'s in distances, angles and torsion angles; correlations between e.s.d.'s in cell parameters are only used when they are defined by crystal symmetry. An approximate (isotropic) treatment of cell e.s.d.'s is used for estimating e.s.d.'s involving l.s. planes.
Refinement. Refinement of *F*^2^ against ALL reflections. The weighted *R*-factor *wR* and goodness of fit *S* are based on *F*^2^, conventional *R*-factors *R* are based on *F*, with *F* set to zero for negative *F*^2^. The threshold expression of *F*^2^ > σ(*F*^2^) is used only for calculating *R*-factors(gt) *etc*. and is not relevant to the choice of reflections for refinement. *R*-factors based on *F*^2^ are statistically about twice as large as those based on *F*, and *R*- factors based on ALL data will be even larger.

### Fractional atomic coordinates and isotropic or equivalent isotropic displacement parameters (Å^2^)

**Table:**

	*x*	*y*	*z*	*U*_iso_*/*U*_eq_	Occ. (<1)
C1	0.79760 (18)	0.61024 (12)	0.64282 (15)	0.0335 (3)	
C2	0.67988 (19)	0.60308 (14)	0.52567 (16)	0.0376 (4)	
H2	0.5718	0.5929	0.5224	0.045*	
C3	0.72677 (19)	0.61135 (13)	0.41723 (15)	0.0363 (4)	
C4	0.8828 (2)	0.62849 (14)	0.41992 (16)	0.0389 (4)	
C5	0.9996 (2)	0.63636 (16)	0.53155 (17)	0.0459 (4)	
H5	1.1066	0.6488	0.5329	0.055*	
C6	0.9547 (2)	0.62527 (14)	0.64410 (17)	0.0396 (4)	
H6	1.0337	0.6281	0.7235	0.048*	
O1	0.63513 (15)	0.60420 (12)	0.29353 (11)	0.0499 (3)	
C7	0.7408 (2)	0.61449 (15)	0.21584 (17)	0.0429 (4)	
H7A	0.7070	0.6739	0.1551	0.052*	
H7B	0.7407	0.5485	0.1666	0.052*	
O2	0.89564 (15)	0.63434 (12)	0.29772 (12)	0.0513 (4)	
C8	0.75138 (18)	0.60240 (12)	0.76304 (15)	0.0335 (3)	
N1	0.60559 (16)	0.62379 (11)	0.75398 (13)	0.0359 (3)	
N2	0.55651 (16)	0.61080 (11)	0.86408 (13)	0.0368 (3)	
H1N2	0.5828	0.5535	0.9113	0.055*	
C9	0.8720 (2)	0.57270 (15)	0.88417 (17)	0.0433 (4)	
H9A	0.8400	0.6009	0.9568	0.065*	0.50
H9B	0.9750	0.6025	0.8843	0.065*	0.50
H9C	0.8802	0.4952	0.8907	0.065*	0.50
H9D	0.9568	0.5315	0.8644	0.065*	0.50
H9E	0.8218	0.5299	0.9370	0.065*	0.50
H9F	0.9166	0.6372	0.9305	0.065*	0.50
C10	0.42636 (18)	0.66446 (12)	0.87292 (15)	0.0328 (3)	
N3	0.37103 (17)	0.73745 (11)	0.78479 (14)	0.0407 (3)	
H1N3	0.4212	0.7432	0.7257	0.061*	
S1	0.34744 (5)	0.63743 (3)	0.99435 (4)	0.03782 (15)	
C11	0.2532 (2)	0.81607 (15)	0.79064 (19)	0.0491 (5)	
H11A	0.2636	0.8777	0.7387	0.074*	0.25
H11B	0.2690	0.8384	0.8792	0.074*	0.25
H11C	0.1473	0.7854	0.7579	0.074*	0.25
H11D	0.1896	0.7900	0.8452	0.074*	0.75
H11E	0.1842	0.8292	0.7047	0.074*	0.75
H11F	0.3059	0.8823	0.8260	0.074*	0.75

### Atomic displacement parameters (Å^2^)

**Table:**

	*U*^11^	*U*^22^	*U*^33^	*U*^12^	*U*^13^	*U*^23^
C1	0.0351 (8)	0.0332 (8)	0.0342 (8)	0.0027 (6)	0.0129 (6)	0.0018 (6)
C2	0.0319 (8)	0.0451 (9)	0.0384 (9)	0.0008 (6)	0.0142 (7)	0.0023 (7)
C3	0.0353 (8)	0.0395 (8)	0.0343 (8)	0.0004 (6)	0.0098 (6)	−0.0001 (6)
C4	0.0409 (9)	0.0447 (9)	0.0355 (8)	−0.0020 (7)	0.0179 (7)	0.0002 (7)
C5	0.0339 (8)	0.0634 (12)	0.0439 (10)	−0.0059 (8)	0.0164 (7)	−0.0005 (8)
C6	0.0346 (8)	0.0483 (9)	0.0369 (9)	−0.0010 (7)	0.0114 (7)	0.0003 (7)
O1	0.0403 (7)	0.0789 (9)	0.0314 (6)	−0.0050 (6)	0.0111 (5)	−0.0002 (6)
C7	0.0473 (10)	0.0492 (10)	0.0354 (9)	−0.0048 (8)	0.0167 (7)	−0.0046 (7)
O2	0.0435 (7)	0.0788 (10)	0.0360 (7)	−0.0057 (6)	0.0182 (6)	0.0010 (6)
C8	0.0354 (8)	0.0328 (7)	0.0343 (8)	0.0018 (6)	0.0129 (6)	0.0027 (6)
N1	0.0384 (7)	0.0407 (7)	0.0326 (7)	0.0052 (6)	0.0165 (6)	0.0050 (5)
N2	0.0391 (7)	0.0416 (7)	0.0334 (7)	0.0071 (6)	0.0164 (6)	0.0084 (6)
C9	0.0388 (9)	0.0531 (10)	0.0379 (9)	0.0000 (7)	0.0101 (7)	0.0081 (7)
C10	0.0328 (7)	0.0335 (7)	0.0327 (8)	−0.0019 (6)	0.0102 (6)	−0.0010 (6)
N3	0.0420 (8)	0.0443 (8)	0.0426 (8)	0.0101 (6)	0.0229 (6)	0.0111 (6)
S1	0.0437 (2)	0.0414 (2)	0.0333 (2)	0.00154 (17)	0.01874 (17)	0.00216 (16)
C11	0.0513 (10)	0.0473 (10)	0.0551 (11)	0.0162 (8)	0.0255 (9)	0.0119 (8)

### Geometric parameters (Å, º)

**Table:**

C1—C6	1.390 (2)	N2—C10	1.355 (2)
C1—C2	1.408 (2)	N2—H1N2	0.8800
C1—C8	1.482 (2)	C9—H9A	0.9800
C2—C3	1.362 (2)	C9—H9B	0.9800
C2—H2	0.9500	C9—H9C	0.9800
C3—O1	1.371 (2)	C9—H9D	0.9800
C3—C4	1.381 (2)	C9—H9E	0.9800
C4—C5	1.362 (3)	C9—H9F	0.9800
C4—O2	1.372 (2)	C10—N3	1.323 (2)
C5—C6	1.399 (2)	C10—S1	1.6935 (16)
C5—H5	0.9500	N3—C11	1.448 (2)
C6—H6	0.9500	N3—H1N3	0.8800
O1—C7	1.427 (2)	C11—H11A	0.9800
C7—O2	1.429 (2)	C11—H11B	0.9800
C7—H7A	0.9900	C11—H11C	0.9800
C7—H7B	0.9900	C11—H11D	0.9800
C8—N1	1.287 (2)	C11—H11E	0.9800
C8—C9	1.496 (2)	C11—H11F	0.9800
N1—N2	1.3957 (18)		
			
C6—C1—C2	119.71 (15)	H9B—C9—H9D	56.3
C6—C1—C8	121.06 (14)	H9C—C9—H9D	56.3
C2—C1—C8	119.22 (14)	C8—C9—H9E	109.5
C3—C2—C1	117.53 (15)	H9A—C9—H9E	56.3
C3—C2—H2	121.2	H9B—C9—H9E	141.1
C1—C2—H2	121.2	H9C—C9—H9E	56.3
C2—C3—O1	127.99 (15)	H9D—C9—H9E	109.5
C2—C3—C4	122.14 (16)	C8—C9—H9F	109.5
O1—C3—C4	109.88 (15)	H9A—C9—H9F	56.3
C5—C4—O2	128.48 (15)	H9B—C9—H9F	56.3
C5—C4—C3	121.85 (16)	H9C—C9—H9F	141.1
O2—C4—C3	109.67 (15)	H9D—C9—H9F	109.5
C4—C5—C6	116.98 (16)	H9E—C9—H9F	109.5
C4—C5—H5	121.5	N3—C10—N2	116.24 (14)
C6—C5—H5	121.5	N3—C10—S1	124.21 (12)
C1—C6—C5	121.76 (16)	N2—C10—S1	119.53 (12)
C1—C6—H6	119.1	C10—N3—C11	124.52 (14)
C5—C6—H6	119.1	C10—N3—H1N3	115.7
C3—O1—C7	106.19 (13)	C11—N3—H1N3	119.1
O1—C7—O2	107.93 (13)	N3—C11—H11A	109.5
O1—C7—H7A	110.1	N3—C11—H11B	109.5
O2—C7—H7A	110.1	H11A—C11—H11B	109.5
O1—C7—H7B	110.1	N3—C11—H11C	109.5
O2—C7—H7B	110.1	H11A—C11—H11C	109.5
H7A—C7—H7B	108.4	H11B—C11—H11C	109.5
C4—O2—C7	106.18 (13)	N3—C11—H11D	109.5
N1—C8—C1	115.47 (14)	H11A—C11—H11D	141.1
N1—C8—C9	124.57 (15)	H11B—C11—H11D	56.3
C1—C8—C9	119.95 (14)	H11C—C11—H11D	56.3
C8—N1—N2	116.66 (13)	N3—C11—H11E	109.5
C10—N2—N1	118.15 (13)	H11A—C11—H11E	56.3
C10—N2—H1N2	117.2	H11B—C11—H11E	141.1
N1—N2—H1N2	120.5	H11C—C11—H11E	56.3
C8—C9—H9A	109.5	H11D—C11—H11E	109.5
C8—C9—H9B	109.5	N3—C11—H11F	109.5
H9A—C9—H9B	109.5	H11A—C11—H11F	56.3
C8—C9—H9C	109.5	H11B—C11—H11F	56.3
H9A—C9—H9C	109.5	H11C—C11—H11F	141.1
H9B—C9—H9C	109.5	H11D—C11—H11F	109.5
C8—C9—H9D	109.5	H11E—C11—H11F	109.5
H9A—C9—H9D	141.1		
			
C6—C1—C2—C3	0.0 (2)	C5—C4—O2—C7	176.38 (19)
C8—C1—C2—C3	179.57 (15)	C3—C4—O2—C7	−2.97 (19)
C1—C2—C3—O1	178.21 (16)	O1—C7—O2—C4	3.96 (19)
C1—C2—C3—C4	−1.4 (3)	C6—C1—C8—N1	157.83 (16)
C2—C3—C4—C5	1.1 (3)	C2—C1—C8—N1	−21.7 (2)
O1—C3—C4—C5	−178.57 (17)	C6—C1—C8—C9	−21.4 (2)
C2—C3—C4—O2	−179.47 (16)	C2—C1—C8—C9	159.10 (16)
O1—C3—C4—O2	0.8 (2)	N1—C8—N1—N2	0 (79)
O2—C4—C5—C6	−178.68 (17)	C1—C8—N1—N2	176.24 (13)
C3—C4—C5—C6	0.6 (3)	C9—C8—N1—N2	−4.6 (2)
C2—C1—C6—C5	1.7 (3)	C8—N1—N2—C10	157.92 (15)
C8—C1—C6—C5	−177.83 (16)	N1—N2—C10—N3	−10.2 (2)
C4—C5—C6—C1	−2.0 (3)	N1—N2—C10—S1	171.13 (11)
C2—C3—O1—C7	−178.00 (18)	N2—C10—N3—C11	−167.92 (17)
C4—C3—O1—C7	1.7 (2)	S1—C10—N3—C11	10.7 (3)
C3—O1—C7—O2	−3.47 (19)		

### Hydrogen-bond geometry (Å, º)

**Table:**

*D*—H···*A*	*D*—H	H···*A*	*D*···*A*	*D*—H···*A*
N3—H1*N*3···N1	0.88	2.17	2.6080 (19)	110
N2—H1*N*2···S1^i^	0.88	2.62	3.4871 (14)	168
N3—H1*N*3···S1^ii^	0.88	2.86	3.4973 (14)	131

Symmetry codes: (i) −*x*+1, −*y*+1, −*z*+2; (ii) *x*, −*y*+3/2, *z*−1/2.
