# Supplementary figures and images for: Crystal structure of (E)-2-[1-(benzo[d][1,3]dioxol-5-yl)ethyl­idene]-N-methyl­hydrazine-1-carbo­thio­amide
Source: Acta Crystallogr E Crystallogr Commun. 2015 Jan 1;71(Pt 1):o35–6. doi: 10.1107/S2056989014026395 (PMC4331916; doi:10.1107/S2056989014026395)

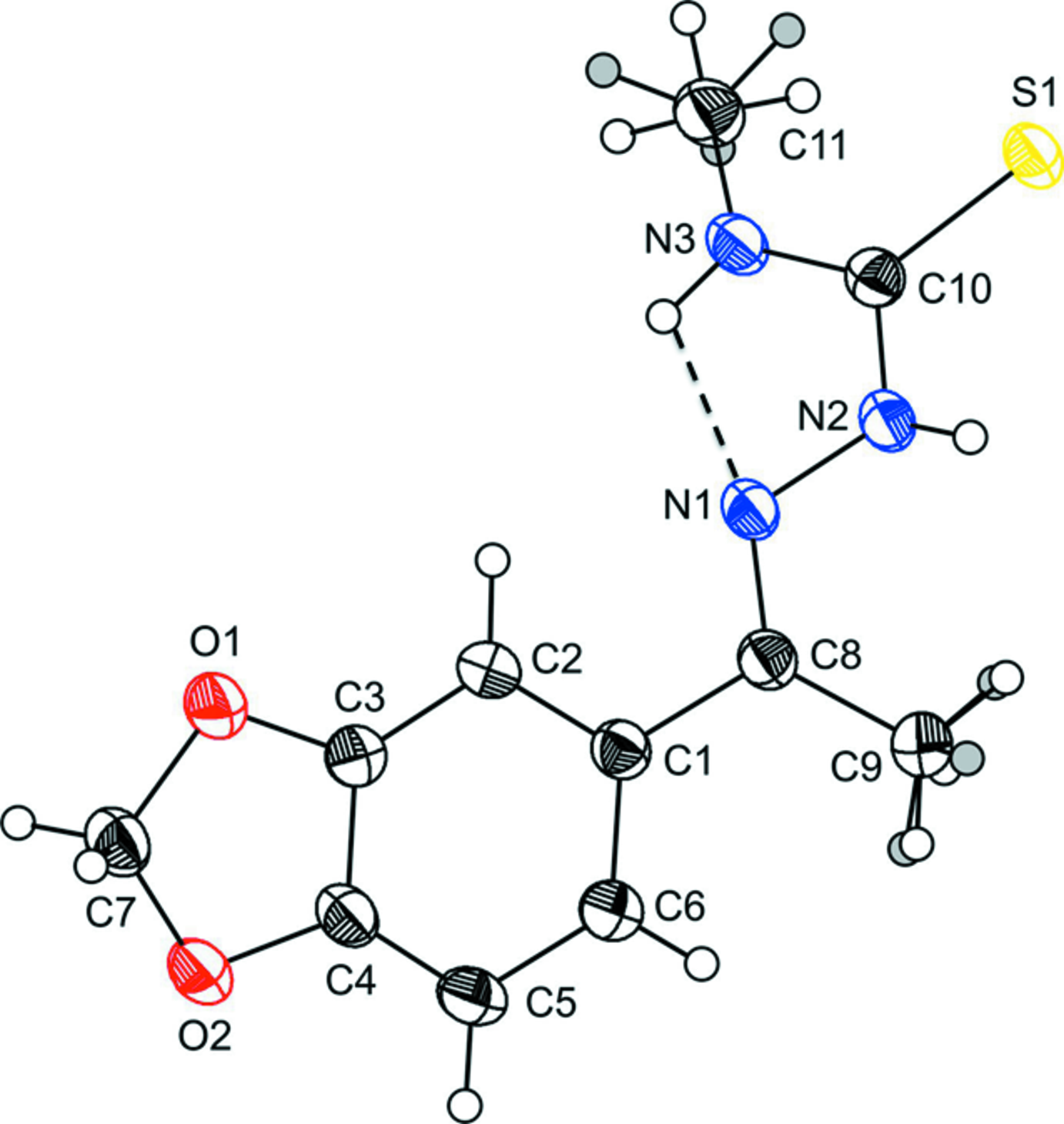

Supplement: Supplementary file 4 [file e-71-00o35-fig1.tif]

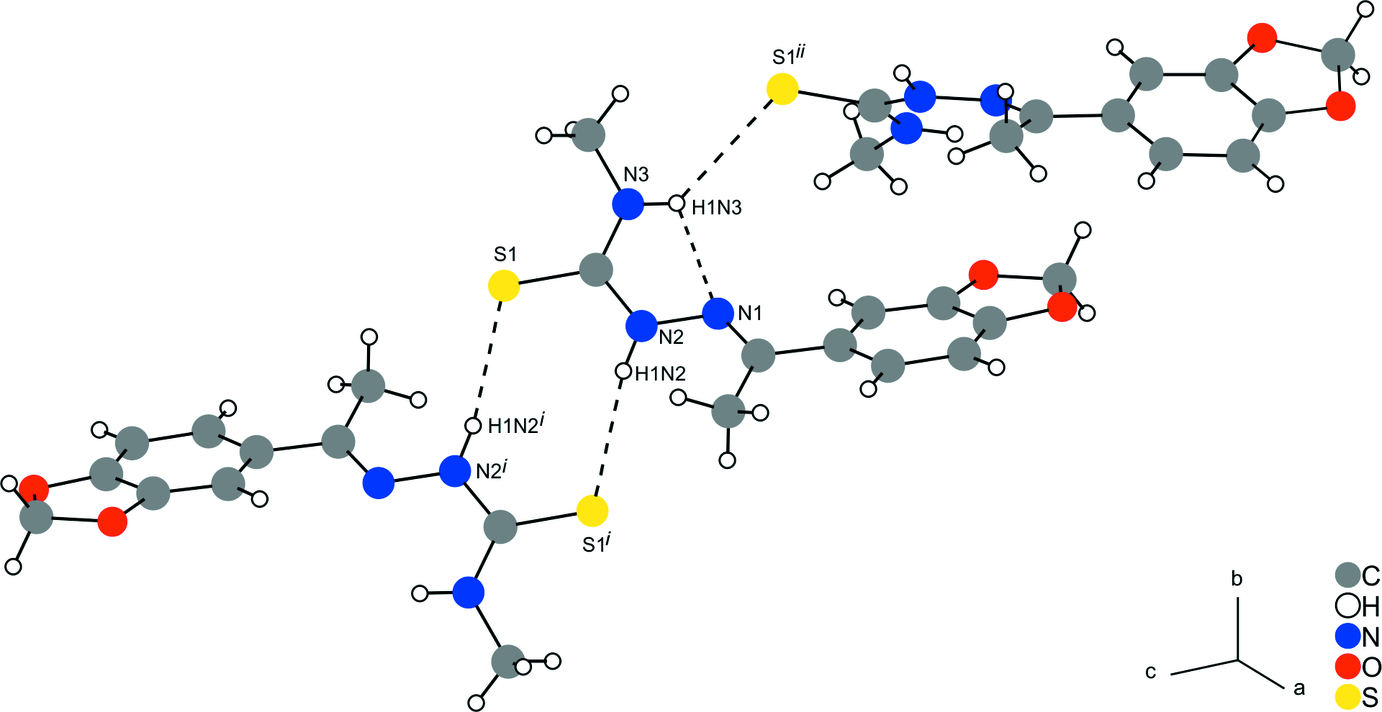

Supplement: Supplementary file 5 [file e-71-00o35-fig2.tif]

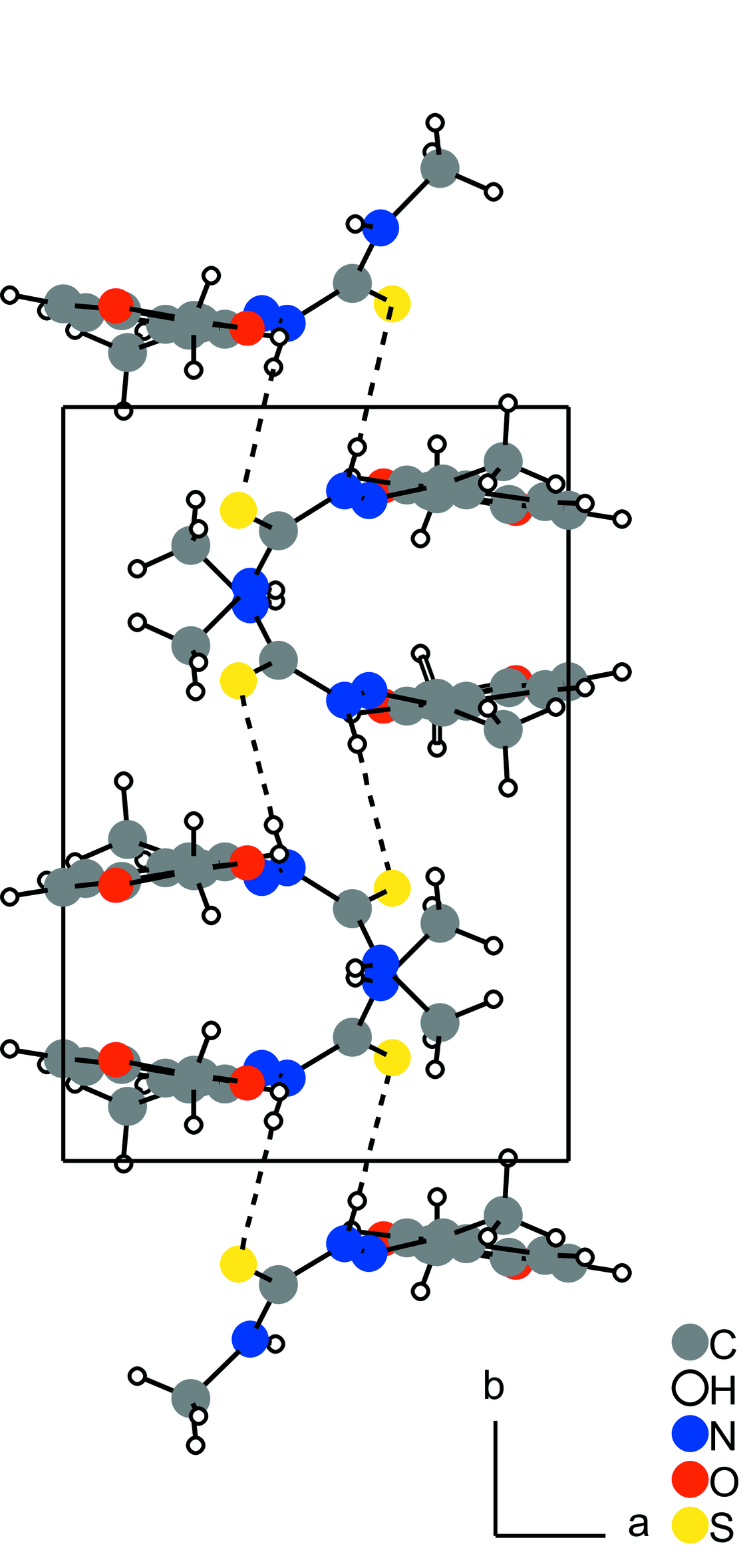

Supplement: Supplementary file 6 [file e-71-00o35-fig3.tif]
